# The Effects of Breastfeeding and Gestational Diabetes Mellitus on Body Mass Composition and the Levels of Selected Hormones after Childbirth

**DOI:** 10.3390/nu15224828

**Published:** 2023-11-18

**Authors:** Dorota Ćwiek, Witold Malinowski, Jarosław Ogonowski, Małgorzata Zimny, Katarzyna Szymoniak, Krystyna Czechowska, Weronika Dawid, Olimpia Sipak-Szmigiel, Grażyna Iwanowicz-Palus

**Affiliations:** 1 Department of Obstetrics and Pathology of Pregnancy, Pomeranian Medical University in Szczecin, 71-210 Szczecin, Poland; dorota.cwiek@pum.edu.pl (D.Ć.); katarzyna.szymoniak@pum.edu.pl (K.S.); krystyna.czechowska@pum.edu.pl (K.C.); weronika.dawid@pum.edu.pl (W.D.); olimpia.sipak.szmigiel@pum.edu.pl (O.S.-S.); 2 Faculty of Health Sciences in Płock, Masovian Public University, 09-402 Płock, Poland; witold05@op.pl; 3 Diabetes Clinic—Independent Public Provincial Integrated Hospital in Szczecin, 71-455 Szczecin, Poland; jogonowski@interia.pl; 4 Department of Specialist Care in Obstetric, Chair of Obstetrics Development of Obstetrics Development, Faculty of Health Sciences, Medical University of Lublin, 20-081 Lublin, Poland; grazyna.iwanowicz-palus@umlub.pl

**Keywords:** pregnancy, body weight, gestational diabetes, BMI, breastfeeding, hormones in breastmilk

## Abstract

Breastfeeding may have a positive effect on glucose metabolism and insulin sensitivity, which may reduce the risk of developing diabetes following gestational diabetes mellitus (GDM). This study aimed to evaluate the effect of breastfeeding and GDM on the body mass composition of the studied women, the levels of leptin, ghrelin, adiponectin, resistin, and insulin, and weight loss during the 6–8-week postpartum period and 1 year after childbirth. Materials and methods: The study group included 42 women with a singleton pregnancy, diagnosed with GDM between the 24th and 28th week of gestation. The control group consisted of 28 non-diabetic women with a singleton pregnancy. This study was carried out at 6–8 weeks as well as at 1 year postpartum. The women were subjected to body weight measurements and body composition analysis performed using a professional body composition analyzer TANITA DC-430 S MA. Waist circumference and subcutaneous fat was measured. Blood for laboratory tests was taken in the morning, on an empty stomach. Results: It was shown that, regardless of diabetes, exclusive breastfeeding had a significant impact on weight loss at 6–8 weeks postpartum (*p* = 0.014785) and lower insulin levels (*p* = 0.047). However, there was no effect of breastfeeding on the women’s anthropometric measurements or hormone levels one year after delivery, except for the thickness of subcutaneous adipose tissue, which was significantly lower in breastfeeding women (*p* = 0.03). One year after delivery, breastfeeding women had a lower BMI (*p* = 0.0014), less-thick subcutaneous adipose tissue (*p* < 0.001), and a lower risk of obesity (*p* = 0.016). There were also higher insulin and ghrelin levels in both breastfeeding and non-breastfeeding women (*p* < 0.001), and lower resistin levels in non-breastfeeding women (*p* = 0.004). Women who had diabetes during pregnancy had a significantly reduced waist circumference and subcutaneous fat thickness after one year (*p* < 0.001 and *p* = 0.05, respectively). Conclusions: Having diabetes during pregnancy did not significantly affect the results of anthropometric measurements and hormone levels noted at 6–8 weeks after delivery (the only exception was the thickness of subcutaneous fat tissue, which was greater in women without GDM). This may indicate normalization of carbohydrate metabolism after childbirth; however, the observation period is too short to elucidate long-term metabolic effects. This suggests the need for further research related to GDM and breastfeeding.

## 1. Introduction

Gestational diabetes mellitus (GDM) is an increasingly common condition worldwide. Women with GDM are at over a 4–7 times higher risk of developing type 2 diabetes after delivery than women with normoglycemic pregnancy [1,2,3]. Approximately 5% of these women will develop type 2 diabetes within the first six months, 10% within 1 to 2 years [4], and nearly 50% within 5–8 years after giving birth [5]. The predictors of diabetes among women with GDM include abnormal glycemia during pregnancy, family history of diabetes, obesity prior to pregnancy, and excessive weight gain during pregnancy or after childbirth [6,7,8]. Therefore, it is important to look for solutions that would reduce the risk of postpartum diabetes and to control its predictive factors. Many authors uphold that exclusive breastfeeding is one of the protective factors. This is because breastfeeding has a positive effect on a mother’s metabolism, insulin sensitivity, a higher rate of glucose disposal, an increased release of fats used in milk production, and thus faster weight loss after delivery [9,10,11,12,13]. Thus, breastfeeding may not only have a short-term effect on postpartum glucose tolerance, but may also delay the development of type 2 diabetes in women with GDM [14]. Among women with pre-existing GDM, breastfeeding had an immediate beneficial effect on glucose tolerance at 4 to 12 weeks postpartum and lower fasting serum glucose levels than non-breastfeeding women [15]. It has been shown that lactation is associated with improved fasting glucose tolerance and increased insulin sensitivity [13]. Breastfeeding also contributes to the lowering of the body mass index (BMI) of lactating women. Women who exclusively breastfed or used mixed feeding with a predominance of breastfeeding had a lower BMI at 6–9 weeks postpartum than non-breastfeeding ones [13]. Breastfeeding was also associated with a long-term reduction in the BMI of postmenopausal women [16].

Adipose tissue is an active endocrine organ that produces hormones—adipokines, which release dozens of different biologically active molecules that affect human metabolism. One of them is leptin. It is a protein produced mainly in subcutaneous adipose tissue by adipocytes, but also by the hypothalamus, pituitary gland, striated muscles, the epithelium of the gastrointestinal tract, as well as by the adipose tissue of the mammary gland, and by placental trophoblast cells [17]. The main role of leptin is to maintain energy homeostasis in the body. To achieve this goal, leptin regulates body fat mass by reducing appetite and food intake and by increasing energy expenditure. Leptin enhances gluconeogenesis and lipolysis in adipose tissue and, consequently, increases blood levels of free fatty acids, decreases lipogenesis and insulin production, and reduces the transport of glucose to adipocytes [18,19].

Another hormone is adiponectin, which is produced and secreted into the blood by mature fat cells. Its source is adipose tissue, and the secretion of adiponectin is stimulated by the insulin produced in the pancreas—by the β cells of the Langerhans islets in response to the increased level of glucose in the body [20]. Adiponectin affects glucose and fatty acid metabolism in the liver and muscles. Its plasma concentration is inversely proportional to the body mass index (BMI), the amount of subcutaneous fat, insulin and triglyceride levels, and directly proportional to the level of HDL cholesterol [21]. The hormone stimulates fat metabolism and increases the sensitivity of liver cells and muscle tissue to insulin [22,23,24]. High levels of adiponectin in the blood are beneficial to health because they result in a reduction in pro-inflammatory cytokines [25] and thus have an anti-inflammatory effect. This hormone also improves insulin sensitivity and increases fatty acid metabolism, and its low level correlates with type 2 diabetes, dyslipidemia, cardiovascular disease, and obesity [24,26]. As such, this hormone may be a marker of insulin sensitivity (its low level is considered an independent risk factor for type 2 diabetes), and it is also useful in determining the risk of cardiovascular disease in humans [17].

Another hormone produced by adipocytes is resistin, which inhibits adipogenesis, i.e., the formation of fat cells (adipocytes). It is involved in the regulation of glucose homeostasis. Rangwala et al. found that high chronic serum resistin levels lead to glucose intolerance and high fasting sugar levels [27]. Resistin levels are higher in obese than lean individuals, and are high in both diet-induced obesity and in genetic obesity and insulin resistance models [28].

Ghrelin is a hormone that causes a rapid increase in food intake and weight gain. It also increases gastrointestinal motility and the secretion of gastric acids. Ghrelin is involved in the short-term regulation of feeding and long-term regulation of energy metabolism, stimulates the secretion of growth hormone (GH) in humans [26], regulates appetite, and is associated with a lower risk of metabolic diseases [29]. Thus, ghrelin may play a role in the regulation of body weight [30]. No Polish studies on this subject have been found.

The study aimed to evaluate the effect of breastfeeding and GDM on women’s body mass composition, selected hormone levels, and weight loss at 6–8 weeks postpartum as well as at 1 year after delivery.

## 2. Materials and Methods

A total of 172 women in the second or third trimester of pregnancy were invited to participate in this study. They were recruited at the Outpatient Clinic for Pregnant Women with GDM and the Specialist State Clinical Hospital No. 1 in Police, Poland. The inclusion criteria for the study group were age over 18 years, singleton pregnancy, and a diagnosis of GDM based on an oral glucose tolerance test (OGTT). GDM was defined on the basis of the guidelines of the Polish Diabetes Association, which are based on the diagnostic criteria for gestational diabetes based on an oral glucose tolerance test with 75 g of glucose according to IADPSG (International Association of the Diabetes and Pregnancy Study Groups, 2010) and WHO (World Health Organization, 2013). An oral glucose tolerance test is performed between the 24th and 28th week of pregnancy in all pregnant women in Poland. Gestational diabetes mellitus (GDM) is diagnosed if at least 1 of the following criteria is met:

- Fasting blood glucose level: 92–125 mg/dL (5.1–6.9 mmol/L);

- Glycemic level 1 h after a 75 g glucose load: ≥180 mg/dL (≥10.0 mmol/L);

- Glycemic level 2 h after 75 g glucose load: 153–199 mg/dL 8.5–11.0 mmol/L) [31].

**Figure 1:**
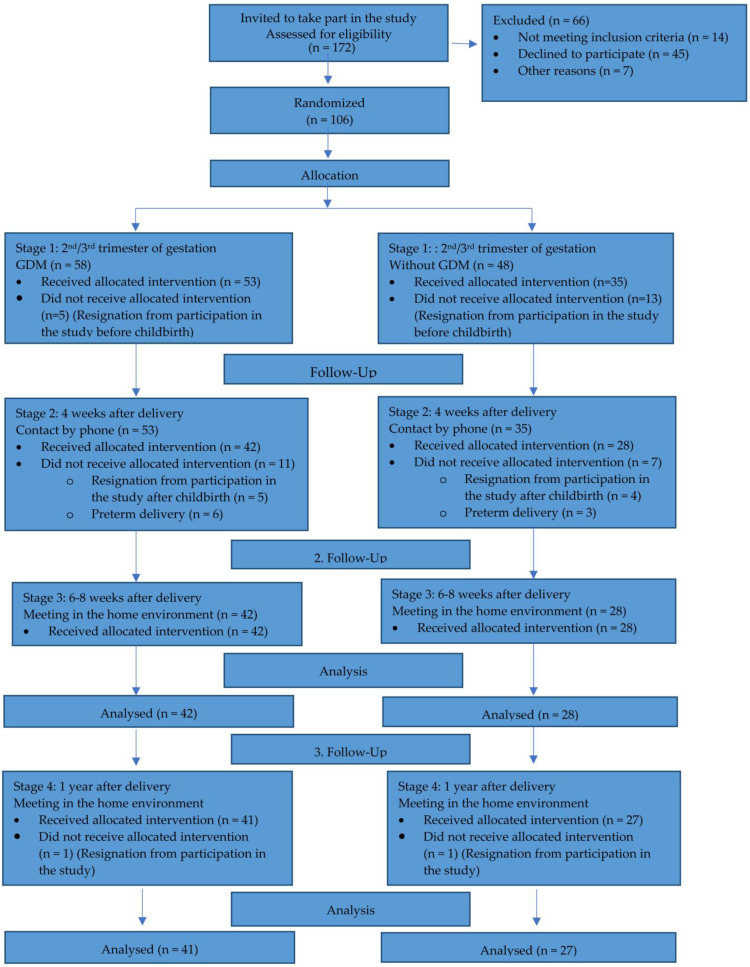
Study profile.

Initially, 106 pregnant women who met the inclusion criteria were enrolled in this study, 66 of whom were excluded based on the following reasons: 45 refused to participate, and 21 were not included for other reasons. The actual examination was carried out 6–8 weeks after delivery. The inclusion criteria for both groups were adjusted for the birth of a baby after 37 weeks of gestation. The women who gave birth before the end of the 37th gestational week were excluded. These criteria were met by 42 women with GDM (study group) and 28 women without it (control group) (60 women). Unfortunately, one year after birth, one patient with GDM and one patient without GDM dropped out of this study (Figure 1). To achieve the aim of this study, the participants were also divided according to the type of baby feeding: exclusive breastfeeding (EBF)—a child fed exclusively with breast milk; mixed feeding (MF)—feeding with breast milk at least four times a day; non-breastfeeding (NBF)—no breast milk provided to the child. After one year, all children had an extended diet, and there was no longer a single child fed exclusively with breast milk.

At the first meeting, the women’s height was measured using a certified medical scale with a height measuring rod. Additionally, an interview was collected from the women regarding sociodemographic data, such as age, education, place of residence, marital status, employment status, and parity. At 6–8 weeks after delivery, during the visit at the patient’s home, birth records of the mother’s weight were analyzed before delivery as well as the baby’s sex and weight at birth. The women were each subjected to a body weight measurement with a body composition analysis (% water, % fat, amount of lean body mass, degree of obesity) using a professional body composition analyzer TANITA DC-430 S MA (Tanita Corporation, Tokyo, Japan). It is certified to be used for medical (professional) use, and meets the NAWI and CLASS III standards for scales used for medical measurements. The analyzer has an EU certification CE0122. With regard to medical devices, it meets the requirements of the Medical Device Directive (MDD) 93/42/EEC. The mothers also had their waist circumference measured with a tailor’s tape halfway between the lower edge of the ribcage arch and the upper edge of the iliac wing, and subcutaneous adipose tissue was measured at the lower angle of the left scapula using a body fat caliper. A Holtain skinfold caliper (GPM) was used to measure skinfold thickness with an accuracy of 0.1 mm. The device is CE certified and complies with the requirements of Directive MDD93/42EEC for medical devices. Additionally, the women’s heights were measured to calculate their BMIs, which were then classified as proposed by the World Health Organization (WHO) [32]. The ideal BMI according to the WHO was defined as 18.5–24.9 kg/m^2^.

One year after delivery, the tests were repeated in the mothers’ home environment. Blood for laboratory tests was taken in the morning (6.00–9.00 a.m) on an empty stomach. After centrifuging the blood at a speed corresponding to 1000× *g* for 15 min at +4 °C, serum was obtained and stored at −70 °C until laboratory analyses were performed. The serum levels of the tested hormones (leptin, ghrelin, adiponectin, resistin, and insulin) were analyzed using the enzyme-linked immunosorbent assay (ELISA) in accordance with the enclosed manufacturer’s instructions. The absorbance measurements were read using the BIOTEC ELx808 (BioTec Instruments, Winooski, USA) plate reader. The tests were performed in the clinical laboratory of the Department of Hypertension, Pomeranian Medical University in Szczecin.

The research was conducted from January 2017 to December 2019. The research protocol was approved by the Bioethics Committee of the Pomeranian Medical University in Szczecin (no. KB-0012/75/2015 of 22 June 2015 and KB-0012/61/2018 of 23 April 2018).

### Statistical Analysis

All data were analyzed using licensed software Statistica 13 (StatSoft Inc. Tulsa, OK, USA). The study sample was described in terms of medians, quartiles, numbers, and percentages. The Shapiro–Wilk test was used to assess the normality of the distribution. The evaluation of the data in the two groups was performed using the Mann–Whitney U test. A comparison of the variables of six weeks postpartum and one year postpartum was performed using the Wilcoxon test. The chi-squared test and the Yates correction were used to analyze qualitative data. The analysis assumed a significance level of 0.05. Thus, all *p*-values below 0.05 were interpreted as indicating significant relationships.

## 3. Results

The mean age of women with GDM was 32.3 years, and 30.57 years of those without diabetes. The majority of the subjects were married (71.4% in the group with GDM and 67.8% without GDM), living in a city (92.9% and 89.3%, respectively), had a good financial status (61.9% and 71.4%, respectively), were employed (83.3% and 71.4%, respectively), and had third-level education (88.1% and 67.9%, respectively). Most women were primiparas (50.0% and 64.3%, respectively). The babies’ average birth weight was 3429.17 g in the group with GDM and 3375.36 g in the group without GDM. The development of diabetes in pregnancy was not found to affect breastfeeding—the difference was not statistically significant at 6–8 weeks after birth (*p* > 0.9088). However, as early as one year after delivery, women with GDM were significantly less likely to breastfeed (39.02% vs. 66.67%, *p* = 0.026). The compared groups did not show statistically significant differences in terms of any of the tested variables. The data are shown in Table 1.

Weight loss at 6–8 weeks postpartum as well as at 1 year postpartum was analyzed in relation to the type of baby feeding and GDM. Exclusive breastfeeding was found to significantly contribute to postpartum weight loss, irrespective of the presence of GDM (*p* = 0.015) (whole group). There were no significant differences in weight loss between mothers with and without GDM 6–8 weeks postpartum as well as 1 year postpartum among mothers who were exclusively breastfeeding, non-breastfeeding, and mixed feeding. It is true that the analysis of body weight one year after delivery showed that breastfeeding non-diabetic women reduced their body weight by 3.9 kg, while breastfeeding diabetic women had a smaller weight loss of 1.6 kg (*p* = 0.413); however, this was not a statistically significant difference. The data are presented in Table 2.

We analyzed the effect of GDM on anthropometric measurements and hormone levels at 6–8 weeks as well as at 1 year postpartum. Women with GDM had a significantly lower subcutaneous adipose tissue thickness 6–8 weeks after delivery (26.0 mm vs. 32.2 mm, *p* = 0.032). The analyses of the other variables showed no differences between the study groups. Also, one year after delivery, no differences were observed between the study groups (Table 3).

We also examined how the parameters studied changed at 6–8 weeks postpartum as well as 1 year postpartum. Women who had diabetes during pregnancy had a significantly reduced waist circumference and subcutaneous fat thickness after one year (*p* < 0.001 and *p* = 0.05, respectively), as well as significantly higher levels of insulin and ghrelin (*p* < 0.001) and lower levels of resistin (*p* = 0.005). Non-diabetic subjects had better anthropometric results one year after delivery. They obtained statistically significantly reduced values for BMI (*p* = 0.002), percentage of body fat (*p* = 0.005), lean body mass (*p* = 0.001), risk of obesity (*p* = 0.002), the thickness of subcutaneous adipose tissue (*p* < 0.001), and waist circumference (*p* = 0.002). They also had higher levels of insulin and ghrelin (*p* < 0.001) (Table 4).

Table 5 shows the effects of the type of baby feeding on BMI, anthropometric dimensions, and hormone levels in both groups. It was observed that at 6–8 weeks after delivery, the levels of insulin in exclusively and partially breastfeeding women were significantly lower than in non-breastfeeding women (3.5 mU/L vs. 5.1 mU/L, *p* = 0.047). The type of feeding had no impact on the remaining variables. One year after delivery, only a significantly lower thickness of subcutaneous fat tissue was observed in breastfeeding compared to non-breastfeeding mothers (20.8 mm vs. 27.9 mm, *p* = 0.030) (Table 5).

Changes in the results of anthropometric measurements and hormone levels in breastfeeding and non-breastfeeding women over a one-year period were analyzed. At 1 year after delivery, breastfeeding women had significantly lower BMIs than at 6–8 weeks postpartum (23.9 vs. 25.4; *p* = 0.014). Moreover, in breastfeeding women, the percentage of obesity significantly decreased after 1 year compared to the period of 6–8 weeks postpartum (8.8 and 15.4, respectively; *p* = 0.016), and so did the thickness of subcutaneous adipose tissue (20.8 mm and 26.8 mm, respectively; *p* = 0.001). In the case of non-breastfeeding women, these relationships were not observed. In contrast, in both breastfeeding (84.0 cm vs. 87.3 cm; *p* < 0.001) and non-breastfeeding (84.8 cm vs. 94.0 cm; *p* < 0.001) women, waist circumference significantly decreased 1 year postpartum compared to 6–8 weeks postpartum.

In our study, 1 year after delivery, significantly higher blood levels of ghrelin were observed in both breastfeeding (161.0 pg/mL vs. 90.5 pg/mL; *p* < 0.001) and non-breastfeeding women (176.5 pg/mL and 96.0 pg/mL; *p* < 0.001) compared to 6–8 weeks postpartum. Similar associations were noted for insulin. Both diabetic and healthy breastfeeding women (5.5 mU/L vs. 2.4 mU/L; *p* < 0.001) and non-breastfeeding women (7.1 mU/L vs. 5.1 mU/L; *p* < 0.001) had significantly higher insulin levels 1 year after delivery than 6–8 weeks postpartum. Resistin levels one year after delivery significantly decreased only in non-breastfeeding women (3.3 ng/mL vs. 3.9 ng/mL; *p* = 0.040) compared to the period just after delivery. The data are presented in Table 6.

Finally, analysis in both groups (with and without GDM) was carried out based on whether BMI, anthropometric measurements, and hormone levels were related to the type of feeding. In the period of 6–8 weeks after delivery, none of the groups showed a correlation between breastfeeding and postpartum body mass composition. Breastfeeding women with GDM had a lower postpartum BMI than breastfeeding healthy ones (23.7 vs. 25.9, respectively; *p* = 0.061) and had a lower degree of obesity; however, the differences were not statistically significant (7.9% vs. 17.5%, respectively; *p* = 0.0606). Perhaps enlarging the study sample would yield statistically significant results. There was also no effect of breastfeeding eight weeks after delivery on the anthropometric measurements in any of the groups. As a matter of fact, breastfeeding women with GDM had a lower subcutaneous fat thickness (26.5 mm vs. 33.7 mm, respectively; *p* = 0.26) and a smaller waist circumference than breastfeeding healthy women (87.0 cm vs. 89.5 cm, respectively; *p* = 0.26); however, the differences were not statistically significant. There were also no differences between groups in leptin, ghrelin, adiponectin, resistin, and insulin levels. Also, one year after delivery, no association was observed between the type of feeding in women with and without GDM and their anthropometric data and hormone levels (Table 7).

## 4. Discussion

Breastfeeding is not only beneficial for the baby, but also for the mother. Greater weight loss after childbirth is observed in breastfeeding women due to higher energy requirements for milk production, and thus the mobilization of fat stores. Our study demonstrated the effect of breastfeeding on weight loss six weeks postpartum in all studied women, regardless of whether the women had GDM or not (*p* = 0.015). However, in a literature review by Gunderson, lactation was not associated with lower weight gain in women who breastfed, compared to those who did not [15]. Butte et al. informed that the mean weight loss during the first six months of lactation in the studied population was about −0.5 to −0.8 kg/month [33]. The reasons for this were attributed to energy expenditure, which was increased by 15–25% for milk production [34]. It should be noted that a woman’s body stores about 4–6 kg of adipose tissue during pregnancy and early lactation for the needs of the fetus and the child after delivery [35]. A study conducted by Rooney et al. on women up to six months after childbirth revealed a correlation between breastfeeding women and weight gain, which was lower than in non-breastfeeding women [36]. It has also been shown that the greater the frequency of feedings, the greater the energy expenditure, which is associated with greater weight loss in the period of three to six months postpartum [37]. Breastfeeding for more than one year resulted in as many as 2 kg greater mean weight loss than in non-breastfeeding women [38], although Olson et al. reported a smaller weight loss of 1.2 kg [37]. In our study, weight loss during the first six weeks after delivery in women with GDM was slightly higher than in those without this condition (10.10 kg vs. 8.58 kg); however, the difference was not statistically significant. Also, one year after delivery, no statistically significant differences in weight loss were observed between breastfeeding women with GDM and healthy ones (0.84 kg vs. 4.31 kg; *p* = 0.143). Palmer et al., on the other hand, noted benefits associated with breastfeeding and faster postpartum weight loss in women with a normal BMI. This relationship was not observed in obese women who had a higher mean weight gain [39]. Vinter et al. noted a negative correlation between exclusive breastfeeding for the first six months after delivery and postpartum weight retention, which contributed to better BMI results [40]. Although breastfeeding may have no effect on short-term weight loss, women who were breastfeeding for at least three months postpartum experienced a significantly lower weight gain at eight years postpartum, and this association was maintained up to 15 years after childbirth [36]. Bobrow et al. found that breastfeeding is associated with a long-term reduction in BMI in postmenopausal women. They showed that the women’s mean BMI decreased by 0.22 kg/m^2^ for every six months of breastfeeding, which corresponded to about a 1% reduction in the mean BMI in the studied population [16]. In our study, no differences were observed between the two groups (GDM and non-GDM) in terms of BMI and body mass composition at either 6–8 weeks or 1 year after delivery. There were also no differences in the thickness of subcutaneous adipose tissue and waist circumference between the groups (GDM and non-GDM) at either 6–8 weeks or 1 year after delivery, irrespective of whether the women were breastfeeding or not. Similar findings can be seen in a literature review by Gunderson, according to whom lactation was not associated with a smaller waist circumference in breastfeeding compared to non-breastfeeding women [15]. On the contrary, Jäger et al. provided evidence that women who never breastfed had higher waist circumference values compared to women who breastfed [41]. Our study showed a significant reduction in waist circumference in all women at 1 year postpartum compared to 6–8 weeks postpartum (*p* < 0.001).

The analysis of body mass composition in terms of the percentage of fat showed that at 6–8 weeks postpartum it was lowest in the group of breastfeeding women with GDM as opposed to the group of breastfeeding women without GDM, where these values were the highest (31.05% vs. 34.74%)—the difference was not statistically significant. In this regard, no relationship was also found between the groups (GDM vs. without GDM) one year after delivery. This is supported by the results obtained by McLachlan et al., showing that the percentage of fat in women with and without GDM was similar [42]. Interesting results were obtained by analyzing anthropometric parameters and hormone levels in mothers with GDM and healthy ones at 6–8 weeks after delivery as well as 1 year after delivery. Better results were observed in mothers without diabetes, whose BMI was significantly reduced and body mass composition values were significantly improved over a year. Some authors indicate that maintaining body weight may be considered one of the risk factors for postpartum diabetes and a sign of worse glucose metabolism [43].

In our study, there were no statistically significant differences in plasma levels of the tested hormones either at 6–8 weeks or 1 year after delivery. Similar conclusions were reached by Chen et al., who noted that there were no significant differences in serum leptin levels between women with and without GDM after delivery [44], although higher levels were observed in women with GDM. Also, in the study by Ranheim et al., leptin concentrations were not significantly different in the two groups (23.3 vs. 22.7 ng/mL, respectively) [45]. Nevertheless, other authors have obtained different results. Kautzky-Willer et al. informed that after delivery, plasma leptin levels were significantly higher (*p* < 0.001) in women with GDM than in women with normal glucose tolerance [46]. McLachlan et al., on the other hand, noticed that postpartum plasma leptin levels were higher in their control group compared to the group with GDM; however, the difference was not statistically significant [42].

Considering plasma adiponectin levels, many researchers uphold that women with GDM have significantly lower levels of this hormone [42,47,48,49,50]. Similar conclusions were drawn by Ranheim et al., who observed that plasma adiponectin levels were lower in GDM patients (8.3 µg/mL) than in controls (11 µg/mL), although this difference did not reach statistical significance, and thus it cannot be excluded as an analysis of adiponectin levels showed a value of 0.53 [45]. In our study, no such relationships were found. In their systematic review, Jäger et al. noted that the longer the mothers breastfed, the higher their adiponectin levels were [41]. In our study, adiponectin was at a similar level in breastfeeding and non-breastfeeding women.

Many authors have reported significantly higher insulin levels in their GDM group than in their control group [44,45,46], which was not confirmed in our study. Considering natural feeding, Tigas et al. noted that breastfeeding women had lower blood glucose and insulin levels compared to non-breastfeeding ones [51], which was not confirmed by our study. However, when analyzing the relationship between the time elapsed after delivery and insulin levels, we found that both breastfeeding and non-breastfeeding women had significantly higher plasma insulin levels at 1 year (*p* < 0.001) than at 6–8 weeks after delivery. In her review of the literature, Gunderson noted that lactation was associated with decreased fasting insulin levels in women who breastfed compared to those who did not. Unfortunately, we obtained different results with regard to breastfeeding [15].

In our study, comparable resistin levels were observed in women with and without GDM. A different observation was made by Megia et al., who reported lower resistin levels in women with GDM than in their non-GDM counterparts. Moreover, resistin levels declined after childbirth, supporting the conclusion that resistin plays a role in the development of diabetes [52]. Indeed, the analysis of the change in resistin levels over a one-year period showed that they decreased significantly in subjects with GDM, which was not observed in those without GDM.

In a study of women at 4–5 weeks postpartum, Larson-Meyer et al. found higher fasting ghrelin levels in breastfeeding mothers than in non- breastfeeding ones (971.8 vs. 798.8 pg/mL, respecitvely) [53]. The analysis of plasma ghrelin levels in our study did not demonstrate any significant differences between the groups (with and without GDM); however, significantly higher levels of this hormone were observed (*p* < 0.001) in both breastfeeding and non-breastfeeding women at 1 year postpartum compared to those observed at 6–8 weeks postpartum. This is supported by a study by Steube et al., who found that a longer breastfeeding duration was associated with higher levels of ghrelin [54].

## 5. Conclusions

Breastfeeding is not only beneficial for the baby but also for the mother, especially if she is diagnosed with diabetes, including GDM. The analysis conducted at 6–8 weeks after delivery showed that breastfeeding contributed to lower insulin levels, while at 1 year postpartum it only resulted in a lower thickness of subcutaneous fat tissue, which suggests that greater fat metabolism is obtained in breastfeeding rather than in non-breastfeeding women. Having diabetes during pregnancy did not significantly affect the results of the anthropometric measurements and hormone levels noted at 6–8 weeks after delivery (the only exception was the thickness of subcutaneous fat tissue, which was greater in women without GDM). This may indicate normalization of carbohydrate metabolism after childbirth; however, the observation period is too short to elucidate long-term metabolic effects, especially considering that at one year after giving birth, women without GDM had slightly better results in anthropometric measurements than their counterparts with GDM. This suggests the need for further research related to GDM and breastfeeding.

## 6. Limitations

What can be considered a limitation of our study is the homogeneity of the research sample (only women of similar ethnic origin were included). Additionally, we do not have any data regarding glycemic control (i.e., glucose levels or HbA1c) in the one-year period after delivery. The mechanisms of how lactation affects GDM, body weight composition, and BMI need to be better understood.

## Figures and Tables

**Table 1:** Sociodemographic data.

Sociodemographic Data		Whole Group n = 70 (%)	With GDM	Without GDM	*p*
			n = 42 (%)	n = 28 (%)	
Marital status	married	49 (70.0)	30 (71.43)	19 (67.86)	0.7494
	single	21 (30.0)	12 (28.57)	9 (32.14)	
Education	vocational	3 (4.29)	1 (2.38)	2 (7.14)	0.1159
	secondary	11 (15.71)	4 (9.52)	7 (25.00)	
	third-level	56 (80.0)	37 (88.09)	19 (67.86)	
Place of residence	the country	6 (8.57)	3 (7.14)	3 (10.71)	0.6010
	city	64 (91.43)	39 (92.86)	25 (89.29)	
Financial situation	average	8 (11.43)	4 (9.52)	4 (14.29)	0.3561
	good	46 (65.71)	26 (61.90)	20 (71.43)	
	very good	16 (22.86)	12 (28.57)	4 (14.29)	
Employment status	employed	55 (78.57)	35 (83.33)	20 (71.43)	0.3032
	unemployed	15 (21.43)	7 (16.67)	8 (28.57)	
Number of children	1	39 (55.71)	21 (50.00)	18 (64.29)	0.3372
	2	22 (31.43)	16 (38.09)	6 (21.43)	
	3 or more	9 (12.86)	5 (11.90)	4 (14.29)	
Type of baby feeding after 6–8 weeks	breastfeeding	39 (55.71)	23 (54.76)	16 (57.14)	0.9088
	mixed feeding	22 (31.43)	13 (30.95)	9 (32.14)	
	formula feeding	9 (12.86)	6 (14.29)	3 (10.71)	
Type of baby feeding after 1 year ^1^	mixed feeding	34 (50.0)	16 (39.02)	18 (66.67)	0.026 *
	formula feeding	34 (50.0)	25 (60.98)	9 (33.33)	
Sociodemographic and anthropometric data		(Me; Q1–Q3)	(Me; Q1–Q3)	(Me; Q1–Q3)	*p*
Mother’s age	(Me; Q1–Q3)	32.0 (28.0–34.0)	32.0 (28.0–35.0)	32.0 (27.0–34.0)	0.1319
Mother’s body weight before pregnancy [kg]	(Me; Q1–Q3)	65.3 (57.0–75.0)	64.5 (58.0–75.0)	66.0 (56.0–79.0)	0.873
Mother’s BMI before pregnancy [kg/m^2^]	(Me; Q1–Q3)	23.6 (21.9–27.6)	23.6 (22.1–27.9)	23.6 (20.1–27.1)	0.716
Mother’s body weight before childbirth [kg]	(Me; Q1–Q3)	79.5 (68.0–90.0)	75.1 (68.0–90.0)	82.0 (72.0–91.0)	0.396
Mother’s body weight 6–8 weeks after childbirth [kg]	(Me; Q1–Q3)	69.9 (60.0–76.7)	65.9 (59.4–74.9)	72.3 (60.4–78.2)	0.403
Mother’s BMI [kg/m^2^]	(Me; Q1–Q3)	24.8 (22.5–28.0)	24.4 (22.6–26.5)	25.6 (22.5–29.2)	0.527
Ideal BMI (weight) [kg]	(Me; Q1–Q3)	59.9 (57.7–63.6)			0.386
Baby’s birth weight [g]	(Me; Q1–Q3)	3400 (3110–3710)	3425 (3130–3710)	3370 (2950–3770)	0.6235
Baby’s birth weight after 6–8 weeks [g]	(Me; Q1–Q3)	4880 (4560–5290)	4910 (4660–5320)	4850 (4550–5260)	0.468

GDM—graviditas diabetes mellitus; Me—median; Q1—first quartile; Q3—third quartile; *p* *—statistical significance. ^1^ One patient with GDM and one patient without GDM dropped out of this study.

**Table 2:** The effect of breastfeeding on weight loss 6–8 weeks as well as 1 year after delivery.

Type of feeding	6–8 Weeks Postpartum									
	Weight Loss [kg] Whole Group n = 70	A. With GDM n = 42				B. Without GDM n = 28				Weight Loss *p* A/B
		Weight Gain during Pregnancy [kg]	BMI kg/m^2^	Weight [kg]	Weight Loss [kg]	Weight Gain during Pregnancy [kg]	BMI kg/m^2^	Weight [kg]	Weight Loss [kg]	
	Me	Me	Me	Me	Me; Q1–Q3	Me	Me	Me	Me; Q1–Q3	
**EBF**	−9.6	10.0	23.7	64.9	−10.5 (−11.5–(−7.2))	9.0	25.9	73.3	−9.10 (−10.0–(−6.7))	0.214
**MF**	−10.8	11.5	25.8	63.1	−10.6 (−11.4–(−10.4))	15.0	24.5	61.0	−11.0 (−16.0–(−10.7))	0.404
**NBF**	−10.2	11.5	25.6	74.2	−9.9 (−10.7–(−6.9))	21.5	25.3	72.2	−11.9 (−12.6–(−11.1))	0.551
**Total**	−10.45	10.5	24.4	66.0	−10.6 (−11.4–(−9.6))	14.0	25.6	72.3	−9.9 (−11.6–(−8.0))	0.587
***p***	EBF/MF + NBF 0.014785 *				EBF + MF/NBF 0.369				EBF + MF/NBF 0.247	
	**1 Year Postpartum**									
**Type of feeding**	**Weight Loss** **[kg]** **Whole Group** **n = 68**	**C.** **With** **GDM** **n = 41**				**D.** **Without** **GDM** **n = 27**				**Weight Loss** ***p*** **C/D**
		**BMI** **kg/m^2^**		**Weight [kg]**	**Weight Loss [kg]**	**BMI** **kg/m^2^**		**Weight [kg]**	**Weight Loss [kg]**	
	**Me**	**Me**		**Me**	**Me; Q1–Q3**	**Me**		**Me**	**Me; Q1–Q3**	
**EBF**	-	-		-	-	-		-	-	-
**MF**	−2.3	24.5		64.2	−1.6 (−3.2–(+2.2))	23.3		68.1	−3.9 (−7.4–(+0.3))	0.143
**NBF**	−0.15	24.1		64.7	0.10 (−4.0–(+1.5))	24.7		65.4	−2.8 (−6.8–(+0.7)	0.447
**Total**	−1.5	24.4		64.6	−0.9 (−3.3–(+1.5))	23.3		67.0	−3.8 (−7.4–(+0.7))	0.082
***p***	MF/NBF 0.387				MF/NBF 0.689				MF/NBF 0.643	

EBF—exclusive breastfeeding; MF—mixed feeding; NBF—non-breastfeeding; GDM—graviditas diabetes mellitus; Me—median; Q1—first quartile; Q3—third quartile; *p* *—statistical significance.

**Table 3:** The effect of GDM on BMI, postpartum body mass composition, and the levels of selected hormones.

Variable	6–8 Weeks Postpartum						*p*
	GDM			Without GDM			
	Me	Q1	Q3	Me	Q1	Q3	
**BMI [kg/m^2^]**	24.4	21.8	26.8	25.6	22.5	29.2	0.527
**Ideal BMI**	59.9	56.3	62.4	61.4	58.5	63.6	0.386
**% of water (TBW) [%]**	47.2	44.2	51.1	47.0	44.2	50.2	0.568
**% of fat [%]**	33.2	26.6	37.8	33.8	28.0	38.1	0.457
**Lean body mass [kg]**	44.8	42.6	48.2	46.9	43.5	49.1	0.268
**Degree of obesity [%]**	10.8	2.6	20.6	16.2	2.3	32.9	0.527
**Subcutaneous fat thickness [mm]**	26.0	21.5	31.2	32.2	26.0	37.0	0.032 *
**Waist circumference [cm]**	90.0	84.0	94.5	88.0	84.0	97.0	0.787
**Insulin level (mU/L)**	3.9	1.6	6.1	2.7	1.5	5.8	0.461
**Adiponectin level (µg/mL)**	7.9	6.7	8.7	7.5	6.4	8.5	0.255
**Leptin level (ng/mL)**	14.6	9.0	22.7	16.5	9.2	23.0	0.662
**Resistin level (ng/mL)**	4.1	1.9	5.7	4.3	1.8	5.1	0.990
**Ghrelin level (pg/mL)**	86.0	71.0	107.0	81.0	75.0	129.0	0.491
**Variable**	**1 Year Postpartum**						***p***
	**GDM**			**Without GDM**			
	**Me**	**Q1**	**Q3**	**Me**	**Q1**	**Q3**	
**BMI [kg/m^2^]**	24.4	21.8	26.8	23.3	21.1	26.8	0.629
**% of water (TBW) [%]**	47.9	45.0	50.7	48.5	45.4	52.0	0.419
**% of fat [%]**	31.5	26.7	36.5	31.4	25.0	36.6	0.527
**Lean body mass [kg]**	43.9	42.8	47.6	45.6	43.0	48.2	0.387
**Degree of obesity [%]**	11.1	−0.8	21.9	5.9	−4.2	22.0	0.607
**Subcutaneous fat thickness [mm]**	23.0	15.0	33.0	22.0	13.0	34.0	0.629
**Waist circumference [cm]**	85.0	79.0	91.0	83.0	77.5	94.0	0.822
**Insulin level (mU/L)**	6.8	4.2	8.6	5.5	3.9	8.3	0.802
**Adiponectin level (µg/mL)**	8.6	6.7	11.8	7.8	5.6	9.9	0.224
**Leptin level (ng/mL)**	15.6	9.3	28.0	11.4	6.6	27.8	0.445
**Resistin level (ng/mL)**	3.3	2.6	3.9	3.6	2.4	4.2	0.679
**Ghrelin level (pg/mL)**	160.0	122.0	220.0	169.0	140	217.0	0.539

GDM—graviditas diabetes mellitus; TBW—total body water; Me—median; Q1—first quartile; Q3—third quartile; *p* *—statistical significance.

**Table 4:** Changes in BMI, postpartum body mass composition, and the levels of selected hormones at 6–8 weeks as well as 1 year after delivery.

Variable	All Group						*p*
	6–8 Weeks Postpartum			1 Year Postpartum			
	Me	Q1	Q3	Me	Q1	Q3	
**BMI [kg/m^2^]**	24.8	22.5	28.0	24.3	21.7	26.8	0.005 *
**% of water (TBW) [%]**	47.0	44.2	50.5	48.0	45.1	51.3	0.029 *
**% of fat [%]**	33.6	27.5	37.8	31.5	263	36.6	0.014 *
**Lean body mass [kg]**	45.6	43.0	48.8	44.7	42.9	48.1	0.001 *
**Degree of obesity [%]**	12.5	2.3	27.4	10.5	−1.3	21.9	0.005 *
**Subcutaneous fat thickness [mm]**	28.3	23.0	35.0	7.2	6.5	8.8	<0.001 *
**Waist circumference [cm]**	88.3	84.0	95.5	84.8	78.8	91.0	<0.001 *
**Insulin level (mU/L)**	3.7	1.5	6.1	6.2	4.1	8.6	<0.001 *
**Adiponectin level (µg/mL)**	7.7	6.7	8.6	8.2	6.2	10.8	0.515
**Leptin level (ng/mL)**	15.0	9.2	22.9	13.2	8.9	27.9	0.453
**Resistin level (ng/mL)**	4.2	1.9	5.3	3.5	2.6	4.1	<0.001 *
**Ghrelin level (pg/mL)**	86.0	71.0	108.0	165.0	130.0	218.5	<0.001 *
**Variable**	**With GDM**						***p***
	**6–8 Weeks Postpartum**			**1 Year Postpartum**			
	**Me**	**Q1**	**Q3**	**Me**	**Q1**	**Q3**	
**BMI [kg/m^2^]**	24.4	21.8	26.8	24.4	21.8	26.8	0.242
**% of water (TBW) [%]**	47.2	44.2	51.1	47.9	45.0	50.7	0.638
**% of fat [%]**	33.2	26.6	37.8	31.5	26.7	36.5	0.392
**Lean body mass [kg]**	44.8	42.6	48.2	43.9	42.8	47.6	0.169
**Degree of obesity [%]**	10.8	2.6	20.6	11.1	−0.8	21.9	0.237
**Subcutaneous fat thickness [mm]**	26.0	21.5	31.2	23.0	15.0	33.0	0.05 *
**Waist circumference [cm]**	90.0	84.0	94.5	85.0	79.0	91.0	<0.001 *
**Insulin level (mU/L)**	3.9	1.6	6.1	6.8	4.2	8.6	<0.001 *
**Adiponectin level (µg/mL)**	7.9	6.7	8.7	8.6	6.7	11.8	0.403
**Leptin level (ng/mL)**	14.6	9.0	22.7	15.6	9.3	28.0	0.282
**Resistin level (ng/mL)**	4.1	1.9	5.7	3.3	2.6	3.9	0.005 *
**Ghrelin level (pg/mL)**	86.0	71.0	107.0	160.0	122.0	220.0	<0.001 *
**Variable**	**Without GDM**						***p***
	**6–8 Weeks Postpartum**			**1 Year Postpartum**			
	**Me**	**Q1**	**Q3**	**Me**	**Q1**	**Q3**	
**BMI [kg/m^2^]**	25.6	22.5	29.2	23.3	21.1	26.8	0.002 *
**% of water (TBW) [%]**	47.0	44.2	50.2	48.5	45.4	52.0	0.006 *
**% of fat [%]**	33.8	28.0	38.1	31.4	25.0	36.6	0.005 *
**Lean body mass [kg]**	46.9	43.5	49.1	45.6	43.0	48.2	0.001 *
**Degree of obesity [%]**	16.2	2.3	32.9	5.9	−4.2	22.0	0.002 *
**Subcutaneous fat thickness [mm]**	32.2	26.0	37.0	22.0	13.0	34.0	<0.001 *
**Waist circumference [cm]**	88.0	84.0	97.0	83.0	77.5	94.0	0.002 *
**Insulin level (mU/L)**	2.7	1.5	5.8	5.5	3.9	8.3	<0.001 *
**Adiponectin level (µg/mL)**	7.5	6.4	8.5	7.8	5.6	9.9	0.962
**Leptin level (ng/mL)**	16.5	9.2	23.0	11.4	6.6	27.8	0.943
**Resistin level (ng/mL)**	4.3	1.8	5.1	3.6	2.4	4.2	0.075
**Ghrelin level (pg/mL)**	81.0	75.0	129.0	169.0	140	217.0	<0.001 *

GDM—graviditas diabetes mellitus; TBW—total body water; Me—median; Q1—first quartile; Q3—third quartile; *p* *—statistical significance.

**Table 5:** The effect of the type of baby feeding on BMI, postpartum body mass composition, and the levels of selected hormones 6–8 weeks as well as 1 year after delivery.

Variable	Type of Baby Feeding 6–8 Weeks Postpartum									*p*
	Whole Group n = 70			EBF + MF n = 61			NBF n = 9			
	Me	Q1	Q3	Me	Q1	Q3	Me	Q1	Q3	
**BMI** **[mean; kg/m^2^]**	24.8	22.5	28.0	24.6	22.5	28.0	24.9	24.3	26.5	0.648
**% of water (TBW) [mean; %]**	47.0	44.2	50.5	47.3	44.7	50.5	46.3	43.7	48.2	0.330
**% of fat** **[mean; %]**	33.6	27.5	37.8	32.7	27.5	37.2	34.9	31.2	38.5	0.305
**Lean body mass [mean; kg]**	45.6	43.0	48.8	45.0	42.3	48.8	46.3	45.6	47.8	0.216
**Degree of obesity [mean; %]**	12.5	2.3	27.4	11.9	2.3	27.4	13.4	10.4	20.5	0.674
**Subcutaneous fat thickness [mean; mm]**	28.3	23.0	35.0	28.5	24.8	34.8	25.0	21.3	38.0	0.958
**Waist circumference** **[mean; cm]**	88.3	84.0	95.5	88.0	83.5	95.5	94.0	90.5	94.5	0.182
**Insulin level (mU/L)**	3.7	1.5	6.1	3.5	1.2	5.7	5.1	3.7	7.3	0.047 *
**Adiponectin level (µg/mL)**	7.7	6.7	8.6	7.6	6.7	8.7	7.9	7.5	8.5	0.642
**Leptin level (ng/mL)**	15.0	9.2	22.9	14.1	9.0	21.6	22.9	11.7	31.8	0.120
**Resistin level (ng/mL)**	4.2	1.9	5.3	4.3	1.9	5.7	3.9	3.1	5.2	0.732
**Ghrelin level (pg/mL)**	86.0	71.0	108.0	83.0	71.0	107.0	96.0	83.0	115.0	0.539
**Variable**	**Type of Baby Feeding 1 Year after Delivery**									***p***
	**Whole Group** **n = 68**			**MF** **n = 34**			**NBF** **n = 34**			
	**Me**	**Q1**	**Q3**	**Me**	**Q1**	**Q3**	**Me**	**Q1**	**Q3**	
**BMI** **[mean; kg/m^2^]**	24.3	21.7	26.8	23.9	21.1	25.7	24.4	21.8	28.9	0.380
**% of water (TBW) [mean; %]**	48.0	45.1	51.3	47.9	45.4	52.0	48.1	43.0	50.6	0.267
**% of fat** **[mean; %]**	31.5	263	36.6	31.	25.1	35.9	31.5	29.9	35.5	0.278
**Lean body mass [mean; kg]**	44.7	42.9	48.1	44.0	42.8	47.6	44.9	42.9	49.5	0.408
**Degree of obesity [mean; %]**	10.5	−1.3	21.9	8.8	−4.2	17.3	11.1	−0.8	31.5	0.384
**Subcutaneous fat thickness [mean; mm]**	7.2	6.5	8.8	20.8	12.5	27.0	27.9	19.0	36.5	0.030 *
**Waist circumference** **[mean; cm]**	84.8	78.8	91.0	84.0	78.0	89.0	84.8	80.0	96.0	0.249
**Insulin level (mU/L)**	6.2	4.1	8.6	5.5	3.6	7.9	7.1	4.7	8.8	0.191
**Adiponectin level (µg/mL)**	8.2	6.2	10.8	8.5	6.1	12.3	8.0	6.3	10.1	0.466
**Leptin level (ng/mL)**	13.2	8.9	27.9	12.4	7.0	28.9	14.4	9.3	24.4	0.650
**Resistin level (ng/mL)**	3.5	2.6	4.1	3.5	2.6	4.0	3.3	2.6	4.2	0.806
**Ghrelin level (pg/mL)**	165.0	130.0	218.5	161.0	133.0	212.0	176.5	125.0	222.0	0.898

EBF—exclusive breastfeeding; MF—mixed feeding; NBF—non-breastfeeding; TBW—total body water; Me—median; Q1—first quartile; Q3—third quartile; *p* *—statistical significance.

**Table 6:** Changes in BMI, body mass composition, and the levels of selected hormones at 6–8 weeks as well as 1 year after delivery.

Variable	6–8 Weeks Postpartum				1 Year Postpartum				*p*
	Type of Feeding n = 70	Me	Q1	Q3	Type of Feeding n = 68	Me	Q1	Q3	
**BMI** **[kg/m^2^]**	EBF n = 39	24.5	22.50	28.00	-	-	-	-	-
	MF n = 22	25.4	22.2	28.3	MF n = 34	23.9	21.1	25.7	0.014 *
	NBF n = 9	24.9	24.3	26.5	NBF n = 34	24.4	21.8	28.9	0.147
**% of water (TBW) [%]**	EBF n = 39	46.9	44.7	51.2	-	-	-	-	-
	MF n = 22	48.6	44.2	50.2	MF n = 34	47.9	45.4	52.0	0.052
	NBF n = 9	46.3	43.7	48.2	NBF n = 34	48.1	43.0	50.6	0.330
**% of fat** **[%]**	EBF n = 39	34.2	27.3	36.9	-	-	-	-	-
	MF n = 22	30.6	27.7	38.1	MF n = 34	31.6	25.1	35.9	0.052
	NBF n = 9	34.9	31.2	38.5	NBF n = 34	31.5	29.9	35.5	0.174
**Lean body mass [kg]**	EBF n = 39	45.7	43.3	49.6	-	-	-	-	-
	MF n = 22	43.9	41.7	48.3	MF n = 34	44.0	42.8	47.6	0.026 *
	NBF n = 9	46.3	45.6	47.8	NBF n = 34	44.9	42.9	49.5	0.027 *
**Degree of obesity [%]**	EBF n = 39	11.1	2.3	27.0	-	-	-	-	-
	MF n = 22	15.4	0.9	28.7	MF n = 34	8.8	−4.2	17.3	0.016 *
	NBF n = 9	13.4	10.4	20.5	NBF n = 34	11.1	−0.8	31.5	0.169
**Subcutaneous fat thickness [mm]**	EBF n = 39	29.5	22.0	35.5	-	-	-	-	-
	MF n = 22	26.8	25.0	34.0	MF n = 34	20.8	12.5	27.0	0.001 *
	NBF n = 9	25.0	21.3	38.0	NBF n = 34	27.9	19.0	36.5	0.069
**Waist circumference** **[cm]**	EBF n = 39	88.5	83.5	96.5	-	-	-	-	-
	MF n = 22	87.3	84.0	95.0	MF n = 34	84.0	78.0	89.0	<0.001 *
	NBF n = 9	94.0	90.5	94.5	NBF n = 34	84.8	80.0	96.0	<0.001 *
**Insulin level (mU/L)**	EBF n = 39	3.6	1.5	5.8	-	-	-	-	-
	MF n = 22	2.4	1.2	5.7	MF n = 34	5.5	3.6	7.9	<0.001 *
	NBF n = 9	5.1	3.7	7.3	NBF n = 34	7.1	4.7	8.8	<0.001 *
**Adiponectin level (µg/mL)**	EBF n = 39	7.6	6.3	9.3	-	-	-	-	-
	MF n = 22	8.0	7.4	8.7	MF n = 34	8.5	6.1	12.3	0.309
	NBF n = 9	7.9	7.5	8.5	NBF n = 34	8.0	6.3	10.1	0.857
**Leptin level (ng/mL)**	EBF n = 39	12.5	8.1	20.2	-	-	-	-	-
	MF n = 22	16.1	10.5	23.0	MF n = 34	12.4	7.0	28.9	0.174
	NBF n = 9	22.9	11.7	31.8	NBF n = 34	14.4	9.3	24.4	0.823
**Resistin level (ng/mL)**	EBF n = 39	4.3	1.8	5.8	-	-	-	-	-
	MF n = 22	4.0	1.9	5.0	MF n = 34	3.5	2.6	4.0	0.061
	NBF n = 9	3.9	3.1	5.2	NBF n = 34	3.3	2.6	4.2	0.004 *
**Ghrelin level (pg/mL)**	EBF n = 39	81.0	69.0	129.0	-	-	-	-	-
	MF n = 22	90.5	75.0	100.0	MF n = 34	161.0	133.0	212.0	<0.001 *
	NBF n = 9	96.0	83.0	115.0	NBF n = 34	176.5	125.0	222.0	<0.001 *

EBF—exclusive breastfeeding; MF—mixed feeding; NBF—non-breastfeeding; TBW—total body water; Me—median; Q1—first quartile; Q3—third quartile; *p* *—statistical significance.

**Table 7:** The impact of GDM on BMI, postpartum body mass composition, and the levels of selected hormones depending on the type of baby feeding.

Variable	6–8 Weeks Postpartum			*p*	1 Year Postpartum			*p*
	Type of Feeding	With GDM n = 42	Without GDM n = 28		Type of Feeding	With GDM n = 41	Without GDM n = 27	
**BMI** **[Me; kg/m^2^]**	EBF n = 39	23.7	25.9	0.061	-	-	-	0.629
	MF n = 22	25.8	24.5		MF n = 34	24.5	23.2	
	NBF n = 9	25.6	25.2		NBF n = 34	24.1	24.7	
**% of water (TBW)** **[Me; %]**	EBF n = 39	47.3	46.3	0.102	-	-	-	0.418
	MF n = 22	48.5	49.2		MF n = 34	47.5	49.2	
	NBF n = 9	45.0	45.8		NBF n = 34	46.3	47.5	
**% of fat** **[Me; %]**	EBF n = 39	32.2	35.3	0.103	-	-	-	0.527
	MF n = 22	30.7	29.2		MF n = 34	32.0	30.3	
	NBF n = 9	36.7	35.3		NBF n = 34	31.0	31.8	
**Lean body mass [Me; kg]**	EBF n = 39	44.8	48.2	0.400	-	-	-	0.387
	MF n = 22	43.3	45.6		MFn = 34	43.8	46.9	
	NBF n = 9	46.9	46.6		NBF n = 34	44.7	45.0	
**Degree of obesity [Me; %]**	EBF n = 39	7.9	17.5	0.061	-	-	-	0.603
	MF n = 22	17.4	11.4		MF n = 34	11.4	5.6	
	NBF n = 9	16.5	14.8		NBF n = 34	9.8	12.4	
**Subcutaneous fat thickness [Me; mm]**	EBF n = 39	26.5	33.7	0.260	-	-	-	0.629
	MF n = 22	26.0	28.0		MF n = 34	19.6	22.0	
	NBF n = 9	23.8	36.0		NBF n = 34	28.0	22.5	
**Waist circumference** **[Me; cm]**	EBF n = 39	87.0	89.5	0.263	-	-	-	0.817
	MF n = 22	88.0	87.0		MF n = 34	85.5	83.0	
	NBF n = 9	94.3	93.5		NBF n = 34	85.0	84.5	
**Insulin level** **[Me; mU/L]**	EBF n = 39	3.9	3.0	0.885	-	-	-	0.797
	MF n = 22	3.7	1.8		MF n = 34	5.4	5.5	
	NBF n = 9	5.6	4.05		NBF n = 34	7.1	5.8	
**Adiponectin level** **[Me, µg/mL]**	EBF n = 39	7.9	7.1	0.685	-	-	-	0.222
	MF n = 22	7.8	8.1		MF n = 34	10.8	7.9	
	NBF n = 9	8.3	7.15		NBF n = 34	8.1	7.8	
**Leptin level** **[Me; ng/mL]**	EBF n = 39	11.9	14.9	0.167	-	-	-	0.441
	MF n = 22	16.10	16.0		MF n = 34	15.7	11.5	
	NBF n = 9	25.0	24.1		NBF n = 34	15.6	11.4	
**Resistin level** **[Me; ng/mL]**	EBF n = 39	4.3	4.1	0.751	-	-	-	0.675
	MF n = 22	3.7	4.7		MF n = 34	3.0	3.6	
	NBF n = 9	3.7	3.7		NBF n = 34	3.3	3.0	
**Ghrelin level** **[Me; pg/mL]**	EBF n = 39	79.0	87.0	0.346	-	-	-	0.535
	MF n = 22	92.0	79.0		MF n = 34	157.0	162.0	
	NBF n = 9	94.0	89.5		NBF n = 34	168.0	177.0	

EBF—exclusive breastfeeding; MF—mixed feeding; NBF—non-breastfeeding; TBW—total body water; Me—median.

## Data Availability

No new data were created or analyzed in this study. Data sharing is not applicable to this article.
